# Polyelectrolyte-Coated Gold Nanoparticles: The Effect of Salt and Polyelectrolyte Concentration on Colloidal Stability

**DOI:** 10.3390/polym10121336

**Published:** 2018-12-03

**Authors:** Melanie Fuller, Ingo Kӧper

**Affiliations:** Flinders Institute for Nanoscale Science and Technology, College for Science and Engineering, Flinders University, Bedford Park 5042, South Australia; melanie.fuller@flinders.edu.au

**Keywords:** polyelectrolyte, stability, gold nanoparticles, gold, biomedical

## Abstract

Gold nanoparticles are widely used in biomedical applications. Their ease of surface modification, biocompatibility and the presence of surface plasmons makes them ideal tools for a variety of investigations. Polyelectrolyte-coated gold nanoparticles are employed in areas such as imaging, drug delivery and gene therapy; however, it is not well understood how different factors such as the polyelectrolyte and salt concentration affect the coating on the nanoparticles and hence their performance. Here, these parameters were systematically varied and their effect on the stability of the colloidal nanoparticle suspension was monitored. An increase in the polyelectrolyte concentration from 0 to 30 mg/mL led to a red shift of the surface plasmon peak and an increase in the zeta potential. Concentrations between 5 mg/mL and 30 mg/mL resulted in the most stable systems, with 1 mg/mL being the most unstable. Stable nanoparticle suspensions were formed in salt concentrations below 50 mM, while higher concentrations caused colloidal instability and irreversible aggregation.

## 1. Introduction

Surface modified gold nanoparticles (AuNPs) are becoming more frequently used in biomedical applications and thus being able to understand the effects various surface coatings have on these NPs is becoming more important [1,2]. AuNPs are being utilised because they are biocompatible and their optical and physical properties make them suitable for sensing purposes. The presence of surface plasmons allows changes in the local environment of the particle to be determined, and the ability to functionalise the surface easily with a range of moieties including polymers, proteins, DNA and polyelectrolytes makes AuNPs suitable for various applications.

**Figure 1:**
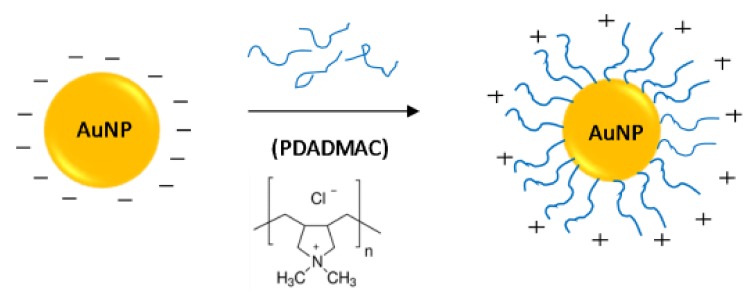
Layer-by-layer (Lbl) attachment of polydiallyldimethylammonium chloride (PDADMAC) onto citrate-capped gold nanoparticles.

The layer-by-layer (Lbl) method of coating NPs and planar substrates using polyelectrolytes (PEs) is well established [3,4,5]. Lbl coatings with PEs have distinct advantages over other surface modification methods, specifically the ease of assembly on a wide range of substrates. The Lbl method allows the sequential addition of oppositely charged PEs onto a substrate through primarily electrostatic interactions (Figure 1) [6]. Polyanions and polycations can be used alternatively to build up multilayer systems which can be used in applications ranging from water treatment to protein immobilisation [7,8]. The ease of deposition of PEs onto NP surfaces is a result of the simple electrostatic interactions that govern the Lbl attachment process. These interactions depend on the type, length and concentration of the PE, as well as the concentration and type of salt used during the assembly process [9]. These variables need to be optimised, especially when depositing PEs onto NPs, as the colloidal stability of the system can be easily affected. According to the DLVO theory, a colloidal solution requires repulsive forces such as electrostatic or steric stabilisation to prevent aggregation from occurring [10]. Electrostatically stabilised systems have an electric double layer, which is due to the surface charge and solvated ions in solution. This double layer results in inter-particle Coulomb repulsion forces which decay exponentially with the particle to particle distance [11]. The thickness of the double layer (Debye length) can be affected by the salt concentration. Increasing salt concentration leads to an increase in the effective screening of these charges, which causes a decrease in the Debye length and hence in the effective distance of the Coulombic interactions [12]. Steric stabilisation can also occur from the absorption of larger molecules such as PEs onto the particle’s surface [11]. These larger molecules can provide a protective layer around the particle, which can also prevent aggregation. As PE-coated NPs can potentially be used in biomedical applications, the colloidal stability is extremely important, as aggregation can alter their in vitro behaviour such as NP uptake and cytotoxicity as well as in vivo fate including biodistribution [13].

The assembly mechanisms as well as the effect of salt, PE concentration and temperature on the formation of planar PE-films are well understood [14]. However, the coating of highly curved surfaces such as the surface of small NPs has not been as thoroughly researched. This is partially due to the added difficulty of coating a highly curved surface and potential aggregation issues with altering the surface coating of NPs. Nevertheless, understanding how salt and concentration affects PE coatings on NP could allow for their use in a wider range of applications.

Here, the effects of both PE concentration and the salt concentration during layer formation has been explored to determine how they affect the formation of a PE-coating on AuNPs. PE concentrations which are too low can lead to incomplete particle coverage and subsequent aggregation. Concentrations that are too high will lead to particle bridging, especially when higher molecular weight polymers are used. Bridging occurs when the polymers absorb simultaneously on more than one particle, ‘grouping’ the particles together causing aggregation [15]. When salt concentrations are too high, it can lead to screening of interparticle electrostatic repulsions, which are needed to prevent aggregation [16,17]. However, it has been proposed that concentrations that are too low can lead to inflexibility of the PE chains, which wrap around the NP resulting in an inadequate or incomplete coating, similar to low PE concentrations [9].

The coating of nanoparticles with PEs can be described by three different scenarios [16]: (1) an excess of particles leads to partial and ‘patchy’ coatings on the nanoparticles, which often results in an increase in particle-particle attractions and subsequent aggregation; (2) equal charge proportions of colloids and PEs yields coated NPs with an overall charge near the isoelectric point of the PE. This in effect neutralises the charge causing the particles to move closer together and aggregate; (3) an excess of PE results in stable solutions of NPs that are saturated by the PE. This final scenario can be expressed as a ratio of polyelectrolyte chains to nanoparticles (PC/NP) and can be calculated by taking the mass of polymer used and dividing by the polymer mass per chain (polymer M_w_/Avogadro’s number) [18]. That number is then divided the number of nanoparticles to give PC/NP. Previously, ratios of 200–4000PC/NP provided the most stability, with the least aggregation [16].

Here, the adsorption of polydiallyldimethylammonium chloride (PDADMAC) of differing concentrations onto AuNPs has been explored using ultraviolet-visible (UV–vis) spectroscopy and zeta-potential measurements. The PE concentration in which the NPs are most stable has then been used to investigate how the salt concentration affects the electrostatic attachment and colloidal stability. By determining the optimum concentrations for the PE and salt, the PE–AuNP system could be better adapted for use in biomedical applications. The optimised salt and PE concentrations were then used to determine if PDADMAC-coated AuNP are more stable than citrate capped AuNP. This was tested through a variety of solvents including ethanol, 10% Tween20 and PBS.

## 2. Materials and Methods

### 2.1. Polyelectrolyte (PE) Concentration

5 nm diameter AuNPs at a concentration of 8.4 × 10^13^ particles/mL were purchased from NanoComposix (San Diego, CA, USA). 2 mL of this particle solution were then mixed with 3 mL of PDADMAC (Sigma Aldrich, Castle Hill, Australia), average Mw < 100,000) solution with concentrations of 0, 0.1, 1, 5, 10 or 30 mg/mL. The AuNP/PDADMAC mixtures were stirred overnight before centrifuging at 14500 rpm for 40 min. The supernatant was removed and the pellet resuspended in 1 mL of 1 mM NaCl. This washing was repeated three times to remove any excess PDADMAC. The pH of the nanoparticle solution was adjusted to pH = 6 using 1 M HCl and measured using the Mettler Toledo pH Meter. The attachment of the PEs onto the NPs and the subsequent stability of the NPs was determined through the use of UV–vis spectroscopy (Cary 50) in 1 mM NaCl with the wavelength range of 400–800 nm. Zeta potential (Malvern Zetasizer Nano, Malvern Instruments Ltd, Malvern, UK) was measured using flow cells with 1 mM NaCl. All experiments were conducted in triplicate.

### 2.2. Salt Concentration

PDADMAC (average Mw < 100,000) in solution, was diluted to a concentration of 5 mg/mL using differing NaCl concentrations of 0, 0.1, 1, 5, 10 and 30 mg/mL. 3 mL of the PDADMAC solution was added to 2 mL of AuNPs and the AuNP/PDADMAC mixture was stirred overnight before centrifuging at 14,500 rpm for 40 min. The supernatant was removed and the pellet resuspended in 1 mL of the same salt concentration. This washing was repeated three times to remove any excess PDADMAC. The pH was adjusted to pH = 6 as per the polyelectrolyte concentration method above. The attachment and stability was also measured as per the PDADMAC concentration protocol; however, during the zeta potential measurements, the samples were measured in their respective salt concentrations. All experiments were conducted in triplicate.

### 2.3. Solvent Stability

Three mL of 5 mg/mL PDADMAC (average Mw < 100,000) in 1 mM NaCl were stirred for 3 h with 2 mL of 5 nm AuNP solution. After 3 h, the sample was centrifuged at 14,500 rpm for 40 min. The supernatant was removed and the pellet resuspended in 1 mM NaCl. This washing was repeated three times to remove any excess PDADMAC. On the final centrifugation step, 50 μL of the pellet was resuspended in 1 mL of 100% ethanol, 10% Tween20 or PBS in triplicate. Similarly, 50 μL of citrate capped 5 nm AuNP (NanoComposix, San Diego, CA, USA) were added to 1 mL of the same three solvents in triplicate. All solutions were then left for 36 h at ambient temperature before surface plasmon resonance (SPR) peak changes were measured by UV–vis spectroscopy (Cary 50) in their respective solvents. In each case, the UV–vis spectrophotometer was blanked with the solvent being used to ensure the solvent itself did not contribute to changes in the SPR peak.

## 3. Results and Discussion

PDADMAC was chosen to coat the NP as it is a strong, highly charged polyelectrolyte, with one cationic charge group per monomer [19]. The type and length of polyelectrolyte remained unchanged for the duration of the experiment and the ionic strength of the medium was controlled to probe the effect of different PE concentrations on the coating of the AuNPs. The specific Mw of PDADMAC was chosen as the longer chains can more effectively wrap around the AuNP, providing better surface coverage.

### 3.1. Effect of PE Concentration

**Figure 2:**
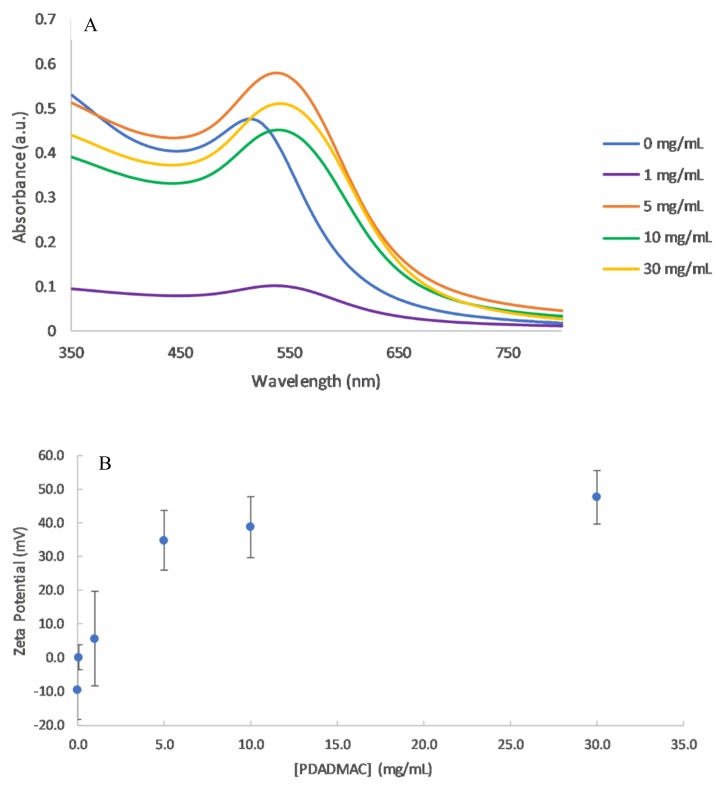
(**A**) UV–vis spectra of PDADMAC coated AuNP at concentrations of 0–30 mg/mL PDADMAC in 1 mM NaCl. (**B**) Zeta potential measurements as a function of PDADMAC concentration at pH 6.0.

In order to investigate the effect of PE concentration on the stability of the colloidal solution, five different concentrations of PDADMAC were used to coat the AuNPs, and the SPR absorbance peaks were measured using UV–vis spectroscopy (Figure 2). Changes in the local nanoparticle environment influence the position of the peak as well as its amplitude. Particle aggregation causes a red-shift in the UV–vis absorption spectrum due to a decrease in interparticle distance [20]. Thus the stability can be monitored through the peak position.

At very low PE concentrations (0.1 mg/mL), the AuNPs fully aggregated after 24 h (Table 1). This concentration corresponds to a PC/NP ratio of 21.5, most likely falling into scenario 2 of near equal charges. This was confirmed through zeta potential measurements, where the cationic PE neutralised the negative charge on the AuNP (Figure 2B). Concentrations below 5 mg/mL have unsaturated layers of PE on the NPs, leading to lower and unstable zeta potentials, as the attractive Van der Waals forces become dominant. Charge neutralisation causes the nanoparticles to move closer together, which results in an unstable suspension and particle aggregation [21]. This is also likely to be the cause for the near zero zeta potential reading in the 1 mg/mL sample, indicating its instability.

Higher concentrations above 5 mg/mL, corresponding to scenario 3 with an excess of PE, led to stable solutions. The PE coatings resulted in shifts in the SPR peak and significantly positive zeta potentials. Above 5 mg/mL, the zeta potential remained relatively stable and this can be attributed to the complete PE coatings on the NPs (Figure 2B). Prior to the plateau in zeta potential, the absorption of PE onto the NP is quantitative, where there would be in effect no free PE still in solution [22].

The absorbance values varied for the different PE concentrations. In principle, the absorbance should be independent of the coating concentration, however particles can be lost through repeated centrifugation wash steps, resulting in lower absorbance values. The lowest absorbance values were observed for 1 mg/mL, where most of the particles aggregated and precipitated out of solution.

The addition of a PDADMAC layer to the AuNP changed the overall dielectric constant of the particles, which resulted in a red shift of the SPR peak. A small change in the SPR peak may also be due to the size of NPs increasing with an additional layer, although this will be minor compared to the change in dielectric constant. The shift was dependent on the PE concentration. For 1 mg/mL PDADAMAC, the SPR shifted by 24 nm (Figure 2A). This shift was much larger than values of 1.5–2 nm reported in other studies [16]. However, both the type and molecular weight of the PE were different in each study which may explain the larger SPR shift. With increasing amounts of the polymer, the SPR peak wavelength increased slowly as not only is the dielectric material surrounding the particle changing, but also the particles are becoming slightly larger with the absorption of the polymer onto the surface [23].

The data collected is in agreement with past studies on PC/NP ratios, where the greatest stability of PE-coated NPs was apparent for PC/NP ratios between 1000–4000 [16]. However, is likely dependent on the structure and Mw of the PE.

### 3.2. Effect of Salt Concentration

**Figure 3:**
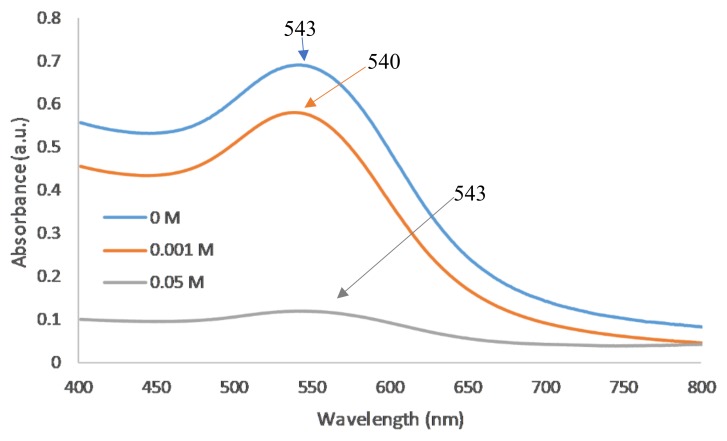
UV–vis spectra of varying salt concentrations of 5 mg/mL PDADMAC on 5 nm AuNPs.

As the likelihood of inter-particle bridging and hence aggregation increases with increasing PE concentration, the lowest stable polymer concentration (5 mg/mL) was used for subsequent salt effect studies. On planar substrates, the maximum coverage of PDADMAC typically increases with increasing ionic strength [24]. For spherical nanoparticles, the absorbance should, thus, increase with increasing salt concentration. However, colloidal stability becomes a greater issue in coating NPs as increasing the salt concentration can increase the Debye screening length. This increased Debye screening length decreases the repulsive electrostatic interactions and hence Van der Waal’s forces dominate causing NP aggregation. Experimentally, an increase in salt concentration caused the NPs to become less stable and at concentrations above 0.05 M, the NPs irreversibly attached to the centrifugation tubes (Table 2). For 0.1 M and 0.5 M NaCl, samples fully aggregated and precipitated. For concentrations of 0.05 M and below, there was no significant difference in peak wavelength (Figure 3) however; the peak absorbance was much lower indicating the nanoparticles have likely started to attach to the centrifuge tubes.

### 3.3. Effect of Solvent

It has been observed that the addition of a PE layer to Au nanorods can increase the stability of NPs in a range of solvents where they otherwise would aggregate [25]. Due to this, the optimised conditions for PDADMAC attachment were used to test the stability of the coated AuNPs in three different solvents: ethanol, Tween20 and phosphate-buffered saline (PBS) to determine if the addition of the PE coating enhanced the NPs stability. The changes were monitored as ΔSPR, which is the difference between the SPR peak in water and the solvent being tested. The larger the shift observed the more likely the NPs are aggregating.

In ethanol, there was a significant shift in the SPR peak, especially for citrate capped AuNPs with ΔSPR = 23 nm, indicating aggregation of the nanoparticles (See supporting information). In comparison, ΔSPR for PDADMAC coated particles was only 6 nm, suggesting a protective effect of the polyelectrolyte coating that reduces the tendency for particle aggregation in ethanol.

Tween 20 had been reported to be a stabilising agent for citrate-capped AuNPs [26,27]. In good agreement with these results, there was no significant difference observed for both citrate-capped and PDADMAC-coated AuNP when exposed to the surfactant.

Finally, PBS was used as it is often used in cell culture studies [28,29]. PBS has a relatively high NaCl concentration (~0.1 M). Similar to the results above, both citrate capped and PDADMAC AuNPs aggregated and attached to the centrifugation tubing, hence no peak could be measured using UV–vis spectroscopy.

## 4. Conclusions

The optimised conditions for polyelectrolyte coatings of 5 nm diameter citrate capped AuNPs were determined to be 5 mg/mL of PDADMAC in 1 mM NaCl. These conditions resulted in the most stable solutions as shown by zeta potential and UV–vis spectroscopy measurements. Additionally, a PC/NP ratio between 1000–4000 resulted in the most stable solutions, in good agreement with the literature. High salt concentrations led to aggregation to the nanoparticles. Finally, when the nanoparticles were suspended in ethanol, the PDADMAC-coated AuNPs showed little aggregation compared to the citrate-coated AuNPs. Further stability testing is required to understand how PDADMAC coatings can prevent aggregation, especially in freeze–thaw experiments. Different polyelectrolytes and nanoparticles will have different influences on the optimised conditions; however, it would be expected that like-sized nanoparticles coated in strong polyelectrolytes would behave in a similar way and, thus, optimisation conditions would be comparable. 

## Figures and Tables

**Table 1:** Concentrations of PDADMAC showing polyelectrolyte chains to nanoparticles (PC/NP) ratio, compared to their average surface plasmon resonance (SPR) peak position and zeta potentials at pH 6.0 ± 0.3.

PDADMAC Concentration (mg/mL)	PC/NP Ratio	SPR Maximum Absorbance Peak (nm)	Absorbance (a.u.)	Zeta Potential (mV)
0	0	513 ± 0.3	0.48 ± 0.2	−9.6 ± 8.9
0.1	21.5	- *	- *	0.3 ± 3.7
1.0	215	537 ± 1.5	0.10 ± 0.09	5.7 ± 14
5.0	1075	539 ± 0.47	0.58 ± 0.04	35 ± 8.9
10	2150	540 ± 2.5	0.45 ± 0.07	39 ± 9.1
30	6450	542 ± 0.94	0.51 ± 0.08	48 ± 7.9

* Fully aggregated and was unable to be removed from the centrifuge tubes with a concentration of AuNP suitable for ultraviolet-visible (UV–Vis) spectroscopy.

**Table 2:** The average SPR peak and absorbance as determined by UV–vis spectroscopy for 5 mg/mL PDADAMAC-coated AuNP.

Salt Concentration (M)	Average SPR Peak (nm)	Average Absorbance (a.u.)
0	543 ± 1.2	0.69 ± 0.04
0.001	539 ± 0.47	0.58 ± 0.04
0.05	540 ± 3.7	0.17 ± 0.05
0.1	*	*
0.5	*	*

* At 0.1 M and 0.5 M, the sample irreversibly aggregated and was unable to be removed from the centrifuge tubes with a concentration of AuNP suitable for UV–vis spectroscopy.

## Supplementary Materials

The following are available online at http://www.mdpi.com/2073-4360/10/12/1336/s1, Table S1: Difference in SPR peak and absorbance for citrate capped AuNP and PDADMAC coated AuNP in Ethanol, Tween20 and PBS.
