# Supplementary material for: Polyelectrolyte-Coated Gold Nanoparticles: The Effect of Salt and Polyelectrolyte Concentration on Colloidal Stability
Source: Polymers (Basel). 2018 Dec 3;10(12):1336. doi: 10.3390/polym10121336 (PMC6402027; doi:10.3390/polym10121336)
Supplement: Supplementary file 1 [file polymers-10-01336-s001.pdf]

Supporting Information

Table S1: Difference in SPR peak and absorbance for citrate capped AuNP and PDADMAC coated AuNP in Ethanol, Tween20 and PBS.

	∆SPR Peak between NPs in MilliQ and NPs in varying solvents (nm)		
Samples	Ethanol	Tween 20	PBS
Citrate Capped AuNP	23 ± 0.38	6 ± 0.31	*
PDADMAC Coated AuNP	6 ± 0.94	2 ± 0.47	*
	Absorbance (a.u.)		
Citrate Capped AuNP	0.149 ± 0.08	0.08 ± 0.008	*
PDADMAC Coated AuNP	0.173 ± 0.001	0.130 ± 0.0009	*

*All samples in PBS irreversibly aggregated and were unable to be removed from the centrifuge tubes with a concentration of AuNP suitable for UV-Vis.
